# Transcriptome sequencing and metabolome analysis to reveal renewal evidence for drought adaptation in mulberry

**DOI:** 10.1049/syb2.70004

**Published:** 2025-02-26

**Authors:** Dan Liu, Changyu Qiu, Sheng Huang, Rongli Mo, Xiaomei Lu, Yanrong Zeng, Guangshu Zhu, Chaohua Zhang, Qiang Lin

**Affiliations:** ^1^ Sericulture Technology Promotion Station of Guangxi Zhuang Autonomous Region Nanning China; ^2^ Guangxi Key Laboratory of Sericultural Genetic Improvement and Efficient Breeding Nanning China

**Keywords:** adaptive control, agricultural engineering, data analysis, genomics, network analysis

## Abstract

As an economically important tree species, mulberry (Morus spp.) has exhibited a remarkable tolerance for salinity, drought and heavy metals. However, the precise mechanism of metabolome‐mediated drought adaptation is unclear. In this study, two new mulberry varieties—‘drought‐sensitive guisangyou62 (GSY62) and highly drought‐tolerant guiyou2024 (GY2024)’—after three days (62F or 2024F) and six days (62B or 2024B) of drought–stress conditions were subjected to transcriptome and metabolome analyses. The enrichment analysis demonstrated that the differentially expressed genes (DEGs) were mainly enriched in carbohydrate metabolism, amino acid metabolism, energy metabolism and secondary metabolite biosynthesis under drought–stress conditions. Notably, compared with the CK group (without drought treatment), 60 and 70 DEGs in GY2024 and GSY62 were involved in sucrose and starch biosynthesis, respectively. The genes encoding sucrose phosphate synthase 2 and 4 were downregulated in GY2024, with a lower expression. The genes encoding key enzymes in starch biosynthesis were upregulated in GY2024 and the transcriptional abundance was significantly higher than in GSY62. These results indicated that drought stress reduced sucrose synthesis but accelerated starch synthesis in mulberry.

## Graphical abstract

In this study, two new mulberry varieties—‘guisangyou62’ (GSY62) and ‘guiyou 2024’ (GY2024) —after three days (62F or 2024F) and six days (62B or 2024B) of drought–stress conditions were subjected to transcriptome and metabolome analyses. The enrichment analysis demonstrated that the differentially expressed genes (DEGs) of the two mulberry varieties were mainly enriched in carbohydrate metabolism, amino acid metabolism, energy metabolism and secondary metabolite biosynthesis under drought–stress conditions. The results indicated that drought stress reduced sucrose synthesis but accelerated starch synthesis in mulberry.

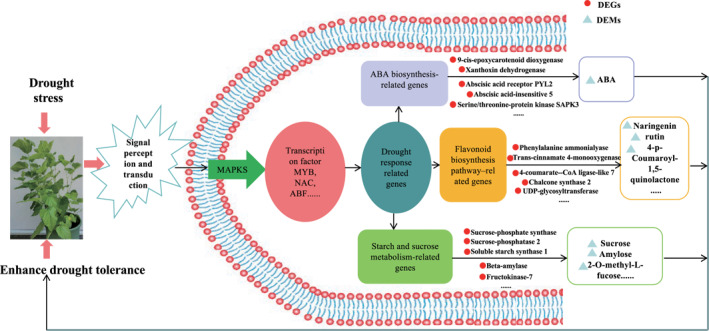

## 1 INTRODUCTION

Mulberry (*Morus* spp.), an important economic crop, has been used as a food for silkworms. It has a long history of cultivation that originated in China [1, 2]. Mulberry is not only rich in nutrients and medicinal ingredients but also exhibits good adaptability to adversity, such as tolerance to salinity, heavy metals, drought and barren conditions [3, 4]. Based on the strong environmental adaptability of mulberry, it can be used for environmental management in ecologically fragile areas, including the management of arid, saline, rocky and desert areas [5, 6]. At present, Guangxi—with typical karst landforms and many rocky desertification areas—has the largest planted area of mulberry trees and water is the main limiting factor of the mulberry yield. Currently, improving the water use efficiency of mulberry trees by genetic methods is a critical challenge in crop breeding research, which requires the systematic elucidation of the underlying molecular mechanisms of drought resistance in mulberry.

Despite the complex molecular mechanisms of drought adaptation in plants, recent advancements in methodological analysis have increased the understanding of drought‐adaptive mechanisms [7, 8]. Drought stress leads to stunted plant growth, reduced yield and even death. During this process, plants develop complex drought tolerance mechanisms to adapt to drought stress. After sensing drought stress signals through receptors on the cell surface, plants produce a secondary messenger (e.g. reactive oxygen species, Ca2+, and phosphoinositide) within the cell. The secondary messenger and different signalling proteins in the cell form a signal transmission chain and amplify the signal through a cascade reaction; this induces the expression of related genes, which encode some regulatory proteins and functional proteins [9].

With the release of the complete genome sequence of *Morus notabilis* [10], there has been increasing functional research on drought‐resistance genes in mulberry [11, 12, 13, 14, 15]. These studies of functional genes have provided a theoretical basis for accelerating the breeding progress of new drought‐resistant varieties of mulberry trees. However, the drought‐adaptive mechanism is not linear but a complex response mechanism involving multiple genes and signalling pathways.

Recently, the development of plant transcriptome and metabolome technologies has provided a new perspective for exploring the molecular mechanisms of plant drought resistance in many species, including millet, cotton, sugarcane and rice [16, 17, 18, 19, 20]. In the research model of ‘metabolome + transcriptome’, the integrated analysis of DEGs and differentially expressed metabolites (DEMs) has been conducted to identify metabolic pathways, hub regulatory factors and structural genes to thereby determine the drought‐adaptive mechanisms [21, 22, 23, 24].

In this study, transcriptome sequencing and metabolic analysis were performed in two mulberry varieties, drought‐sensitive GSY62 and highly drought‐tolerant GY2024. The many DEGs between the two strains were screened by analysing GO and KEGG transcriptomic data and these DEGs, combined with the DEMs in the subsequent analysis, were further analysed. These results lay a foundation for furthering the understanding of the molecular mechanisms of mulberry drought tolerance and have application values for drought land management and drought resistance resource screening.

## 2 MATERIALS AND METHODS

### 2.1 Plant materials, cultivation and drought‐stress experiment

Two mulberry stains are employed in this study—GY2024 and the GSY62. In the winter of 2020, the annual grafted seedlings of GY2024 and GSY62 that were growing well were transplanted into a potted barrel. The potted seedlings were then placed in the same transparent greenhouse, with identical water and fertiliser management in the early stages. The summer pruning was performed at the end of July 2022, with the plants pruned to 30 cm from the substrate surface. In early September 2022, the plants with good growth and consistent upward trends were used for stress treatment. To establish drought–stress conditions, the sample plants of GSY62 and GY2024 were transferred into an 8% PEG‐6000 solution for long‐term treatment. A WP4C dew ‐ point potentiometer (METER, USA) was used to measure the water potential of the 8% PEG6000 solution. Before the start of the experiment and during the experimental process, the water potential of the solution was measured every 24 h to ensure the stability of drought stress. The water potential of this concentration of PEG6000 solution was measured to be approximately 0.5 MPa, which was used as a quantitative indicator of the degree of simulated drought stress. The temperature was set at 25°C; the light intensity was 150 μmol·m⁻^2^ s⁻^1^, and the light duration was maintained at 12 h per day; the relative humidity was maintained at 55%–65%. After 3 d or 6 d of treatment, the leaves of GSY62 and GY2024 were collected and stored in liquid nitrogen for the subsequent omics detection. The groups were established as follows: the GY2024 control group (2024CK) and different drought stress duration groups (2024F and 2024B for three and six days, respectively), the GSY62 control group (62CK) and different stress duration groups (62F and 62B for three and six days, respectively). Each group had 3 biological replicates, and each replicate contained 10 seedlings. The seedlings in the control group were watered with normal MS nutrient solution, while the seedlings in the experimental group were watered with an 8% PEG6000—MS mixed solution to simulate the drought–stress environment.

### 2.2 Transcriptome sequencing and metabolome measurement

For transcriptome and metabolome sequencing, three biological replicates of each group were prepared. TRIzol® reagent was utilised to isolate the total RNA from the leaf tissue according to the manufacturer's instructions. The quality and concentration of total RNA were determined using the 5300 Bioanalyser (Agilent) and ND‐2000 (NanoDrop Technologies), respectively. To construct a reliable sequencing library, 1 μg of high‐quality RNA sample (OD260/280 = 1.8–2.2, OD260/230 ≥ 2.0, RIN ≥6.5, 28S:18S ≥ 1.0, >1 μg) was sequenced on the Illumina NovaSeq 6000 platform (San Diego, CA; Shanghai Majorbio Bio‐pharm Biotechnology Co., Ltd, China). The sequences in the library were prepared at a 2 × 150 bp read length.

For metabolome analysis, the metabolites were extracted from the leaves of drought‐stressed plant samples and then analysed using an LC‐MS/MS system (UHPLC‐Q Exactive HF‐X system, Thermo, USA) with an ACQUITY HSS T3 column (100 mm × 2.1 mm i. d., 1.8 μm; Waters, USA). The mobile phase passed through the column at a flow rate of 0.40 mL·min–1 at 40°C. Subsequently, the mass spectrometric data were collected using a UHPLC‐Q Exactive HF‐X Mass Spectrometer (Thermo, USA). Data were collected in the data dependent acquisition (DDA) mode.

### 2.3 Bioinformatics analysis

The quality control of raw sequencing data was performed using the fastp online tool (https://github.com/OpenGene/fastp). The clean reads were used to map to the reference genome using the Hisat2 online tool (http://ccb.jhu.edu/software/hisat2/index.shtml), producing the reassembled transcriptome data. The “DESeq2” package was used to analyse the DEGs. The cutoff criteria were established as |log2FC| ≥ 1 and FDR <0.05(DESeq2). Additionally, the functional enrichment analysis including GO and KEGG pathway analysis was performed using Goa tools and Python SciPy, respectively.

The original LC/MS data file was imported into Progenesis QI (Waters Corporation, Milford, USA) and then exported as a three‐dimensional data matrix in a CSV format. The metabolites were identified by searching in HMDB (http://www.hmdb.ca/), Metlin (https://metlin.scripps.edu/) and Majorbio databases. Then, the obtained metabolite matrix was submitted to the Majorbio cloud platform (https://cloud.majorbio.com) for subsequent data analysis. Briefly, the “ropls” (Version 1.6.2) package in R Studio was used to conduct the principal component analysis (PCA) and the orthogonal least partial squares discriminant analysis (OPLS‐DA). The variable importance (VIP) obtained from the OPLS‐DA model and the *p*‐value generated by Student's *t*‐test were utilised to evaluate the significance. The cut‐off values were set as VIP >1 and *p* < 0.05. The DEMs of the two groups were submitted into the KEGG database for mapping of their biochemical pathways.

### 2.4 Quantitative PCR (qRT‐PCR)

The RNAiso Plus kit (Takara, Japan) was utilised to isolate the total RNA. Then, 1000 ng of total RNA was used to perform reverse transcription using the Prime Script RT reagent kit (TaKaRa, Japan). 2 μL of the synthesised first‐strand cDNA was used as a template. The SYBR Premix reagent (Toyobo, Japan) was utilised for qRT‐PCR. The reactions were performed on an Applied Biosystems Step One Plus RealTime PCR System (Applied Biosystems, USA). Three independent experiments were conducted and three technical replicates for each sample were required.

## 3 RESULTS

### 3.1 Transcriptome analysis of the GSY62 and GY2024 strains

#### 3.1.1 Transcriptome characteristics and relationship analysis of GSY62 and GY2024 under drought‐stressed conditions

The transcriptome data used in this study were obtained from the drought‐resistant variety GY2024 and the drought‐sensitive variety GSY62 using the next‐generation sequencing platform Illumina Novaseq 6000. After quality control, 115.15 Gb of clean reads were obtained, with each sample having more than 6.28 Gb of clean reads and the Q30 base percentages were all 100% (Table S1). Then, sequence alignment was performed between the clean reads of each sample and the designated reference genome. The alignment rates ranged from 69.4% to 71.94% (Table S2), which satisfied the subsequent annotation analysis requirements. The reads that mapped to the unannotated region of the reference genome gtf file were defined as new genes. A total of 47,404 genes were assembled, including 2945 new genes (Table S3).

As shown in Figure S1a, there were 13,550 common genes in the six groups of samples, accounting for 75.54%. The proportion of unique genes is the highest in PEG_624B, at 1.76%, whereas the proportion of unique genes is the lowest in PEG_62‐F, at only 0.27%. Correlation analysis between biological replicates not only verifies whether the variations between biological replicates meet the expectations of experimental design but also provides a basic reference for differential gene analysis. The closer the correlation coefficient is to 1, the higher the similarity of gene expression levels between samples, that is, the better the correlation between samples. PCA can reduce the complexity of data, so we can use PCA to identify outlier samples and distinguish sample clusters with high similarity. As shown in Figure S1b,c, each group of samples and biologically duplicated samples clustered together, whereas the treatment groups were clustered in different regions, indicating good sample repeatability.

#### 3.1.2 Expression pattern analysis of DEGs in GSY62 and GY2024 under drought stress

**FIGURE 1:**
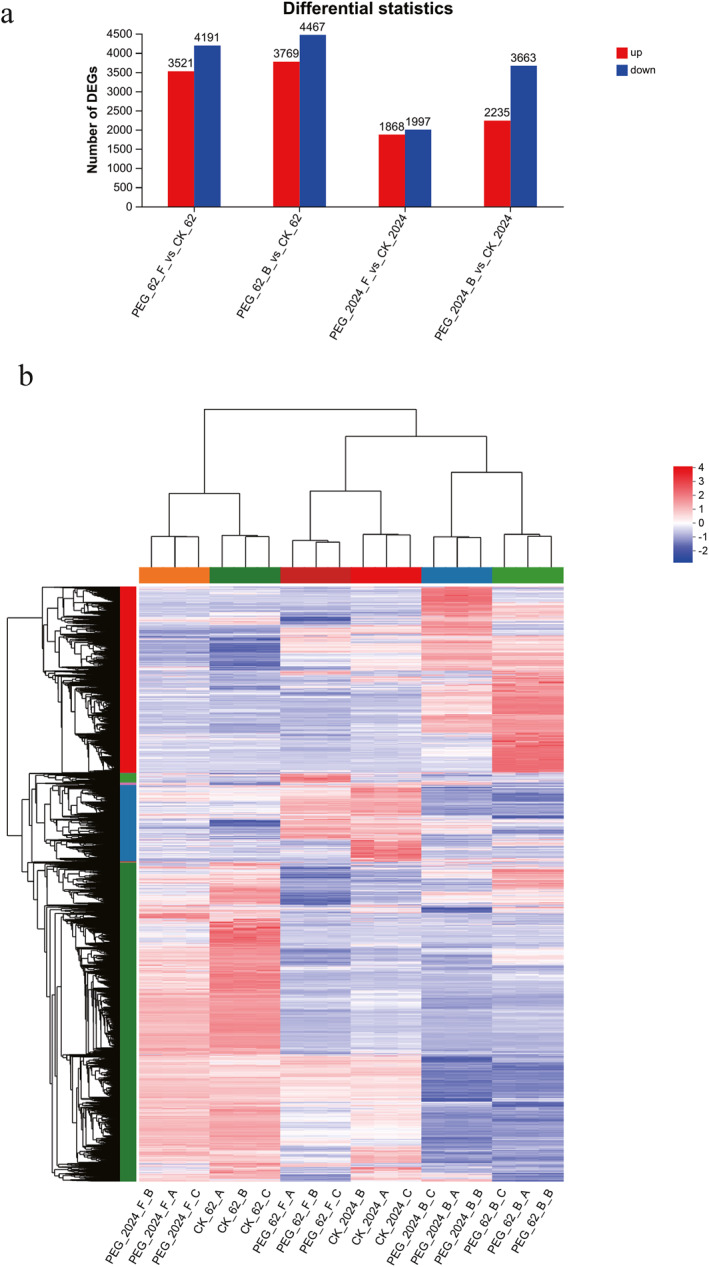
Identification and analysis of DEGs between four comparison groups: 62F vs. CK, 62B vs. CK, 2024F vs. CK and 2024B vs. CK. (a) Number of up‐ and downregulated genes. (b) Cluster heatmap of DEGs in mulberry leaves. Each column in the graph represents a sample, and each row represents a gene. The horizontal axis represents the multiple expression differences of genes/transcripts between two samples, which is the numerical value obtained by dividing the expression level of the treated sample by the expression level of the control sample. The vertical axis represents the statistical test value of the difference in the gene expression level, which is the *p*‐value. The larger the log10 (*p*‐value), the more significant the difference in expression. The values on the horizontal and vertical coordinates have been logarithmised. Each dot in the graph represents a specific gene; red dots represent significantly upregulated genes, green dots represent significantly downregulated genes and grey dots represent non‐significantly DEGs. The closer the points on both sides and above, the more significant the difference in expression. The colours in the graph represent the standardised expression levels of the gene in each sample, with red indicating a higher expression level of the gene in the sample and blue indicating a lower expression level. The specific trend of expression level changes can be found in the numerical annotation below the colour bar in the upper left corner. The left side shows the tree diagram of gene clusters and the module diagram of sub clusters, whereas the right side shows the names of genes. The closer the two gene branches are, the closer their expression levels are. The upper part is a tree diagram of sample clustering, and the lower part is the name of the sample. The closer the branches of the two samples are, the closer the expression patterns of all genes in these two samples are, that is, the closer the trend of gene expression changes is.

The discrepancy in the capability of drought adaptation between mulberry GSY62 and GY2024 may present as differences in transcriptome dynamics (Figure 1a). Compared with the CK group, GSY62 had a total of 7712 DEGs (|log2FC| ≥ 1) and FDR <0.05(DESeq2) after three days of drought stress treatment (62F), consisting of 3521 upregulated and 4191 downregulated genes (Figure 1a), as well as a total of 8236 DEGs after six days (62B), including 3769 upregulated and 4467 downregulated genes (Figure 1a). Compared to GY62, a total of 3865 DEGs in GY2024 (drought resistant type) were identified after three days of drought stress (2024F), including 1868 upregulated and 1997 downregulated genes (Figure 1a), but there were 5898 DEGs after six days of drought stress (2024B), consisting of 2235 upregulated and 3663 downregulated genes (Figure 1a). Detailed information on DEGs, including associated pathway information, statistical significance, and fold‐changes. etc., can be found in Tables [Link], [Link], [Link], [Link]. The total number of DEGs in GY2024 was much lower than that of GSY62 under drought stress. This indicated that drought stress had a smaller impact on GY2024, which coincided with the findings of our previous screening experiments on drought‐tolerant mulberry. Furthermore, there were significantly more downregulated DEGs than upregulated genes in the two strains. These downregulated genes were mostly genes that control the growth and development of mulberry, consistent with symptoms such as wilting and defoliation in mulberry seedlings under drought stress to cope with water scarcity stress.

Cluster analysis is used to determine the expression patterns of DEGs in different samples, clustering genes with similar or identical expression patterns into clusters, and identifying the functions of unknown genes or unknown functions of known genes. These similar genes may have similar functions, participate in the same metabolic process together, or exist in the same cellular pathway. In this study. we extracted the expression levels of DEGs from all samples into a gene set and then plotted the corresponding heatmap. As shown in Figure 1b, drought stress suppressed most of the gene expression in mulberry, especially in the later stages of drought. The function of downregulated genes mainly involved cell membrane components. Moreover, the gene expression patterns induced by drought were also different in GY2024 and GSY62. Among them, some genes in GY2024 were upregulated and induced, while these genes did not show this response in GSY62, including key genes in the abscisic acid signalling pathway and carbohydrate pathway signalling pathway. Therefore, these genes may be related to the drought tolerance of mulberry trees and will be the focus of future research.

#### 3.1.3 Functional enrichment analysis of DEGs in mulberry leaves

**FIGURE 2:**
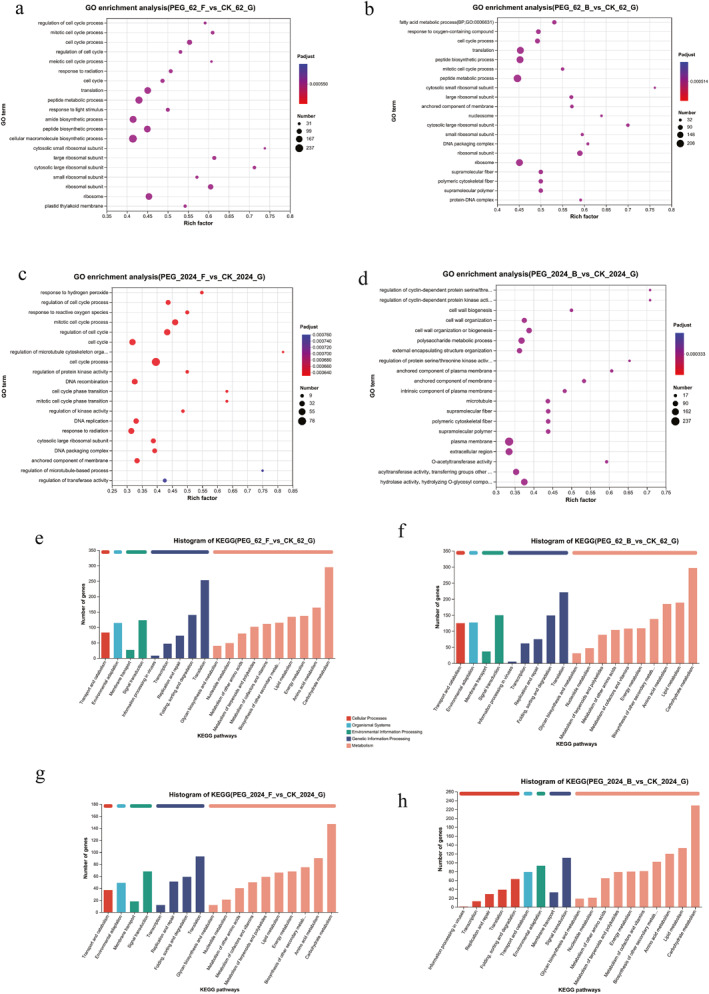
GO and KEGG functional enrichment analysis of DEGs in mulberry leaves. Bubble charts of the top 20 GO entries for the most prominent DEGs between the four comparison groups: (a) 62F vs. CK, (b) 62B vs. CK, (c) 2024F vs CK, (d) 2024B vs. CK. Histogram of KEGG functional classification between the four comparison groups: (e) 62F vs. CK, (f) 62B vs. CK, (g) 2024F vs. CK, and (h) 2024B vs. CK. The vertical axis represents the GO term and the horizontal axis represents the Rich factor [the ratio of the number of genes/transcripts enriched in this GO term to the number of annotated genes/transcripts (background number); the larger the Rich factor, the greater the degree of enrichment]. The size of the dot represents the number of genes/transcripts in the GO term and the colour of the dot corresponds to different Padjust ranges.

To further clarify the function of DEGs, the topGO package (v2.12.0) was used to filter the significant GO entries enriched by the obtained DEGs. The top 20 GO processes or pathways are listed and visualised in Figure 2a–d (*p*‐adjust <0.05). The results demonstrated that the most prominent DEGs identified in the two strains affected several biological processes that have similar GO entries, including translation (GO:0006412), regulation of cell cycle process (GO:0010564), response to reactive oxygen species (GO:000,302) and cell wall biogenesis (GO:0042546). The above data suggested that the two mulberry lines, GSY62 and GY2024, showed a considerable similarity in response to drought stress. However, the polysaccharide metabolism process (GO:0005976) and the regulation of protein serine/threonine kinase activity (GO:0071900) were specifically enriched in GY2024, whereas the peptide metabolism process (GO: 006518) was specifically aggregated in GSY62. These may be unique processes by which different mulberry varieties respond to drought stress.

A KEGG pathway analysis result demonstrated that the DEGs identified in both strains were markedly involved in some similar molecular pathways, including carbohydrate metabolism (such as starch and sucrose metabolism), amino acid metabolism, energy metabolism and secondary metabolite biosynthesis(Figure 2e–h). Notably, the DEGs were more enriched in energy metabolism compared to secondary metabolite biosynthesis in 62F, but the opposite was true in 62B. In contrast, more DEGs were concentrated in secondary metabolite biosynthesis than in energy metabolism in both 2024F and 2024B. More importantly, with the increased duration of drought stress treatment, the number of DEGs aggregated in secondary metabolite biosynthesis increased in both the GY2024 and GSY62 strains, with increases of 26.5% and 16.7%, respectively. These results indicated that under drought stress, GY2024 could accelerate its response to external stimuli by promoting the synthesis of more secondary metabolites. In summary, these mulberry strains mainly adapted to and resisted drought stress by adjusting their internal biosynthesis and metabolism processes. The KEGG analysis results were similar to the GO analysis results, for example, both showed enrichment of the processes related to sugar synthesis metabolism, secondary metabolite synthesis and energy metabolism.

#### 3.1.4 Expression profiling of key metabolism‐related genes

##### Starch and sucrose metabolism‐related genes

**FIGURE 3:**
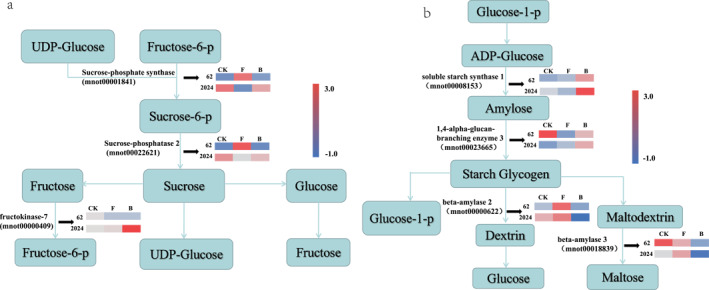
Simplified mechanistic model of the key DEGs in GSY62 and GY2024 and the (a) sucrose and (b) starch metabolism pathways. Each coloured cell represents the average log2 (TPM) value according to the colour scale; red represents high expression and blue represents low expression.

There were significant differences in the expression levels of key enzyme‐related genes involved in sucrose biosynthesis and metabolism between GY2024 and GSY62. After drought stress, the genes encoding sucrose phosphate synthase 4 and sucrose phosphatase 2 were downregulated in GY2024 but upregulated in GSY62. Additionally, the gene encoding fructose kinase‐7 was more upregulated in GY2024 than in GSY62, with an upregulation amount 8 times that of GSY62 (Figure 3a). These results showed that sucrose synthesis was inhibited in GY2024 and fructose production was promoted under drought stress, indicating that under drought stress, drought‐tolerant mulberry GY2024 reduced sucrose synthesis and accelerated sucrose degradation.

There were also significant differences in the expression levels of key enzyme‐related genes involved in starch biosynthesis and metabolism between GY2024 and GSY62. The key enzymes encoding starch biosynthesis—mnot00008153 and mnot00023665—were upregulated in GY2024 and their transcriptional abundance was significantly higher than in GSY62 (Figure 3b). The key enzyme‐related genes encoding dextrin and maltose—mnot00000622 and mnot00018839—were downregulated in GY2024 (Figure 3b). These results indicated that the synthesis and metabolism of starch in mulberry were affected by drought, with drought stress accelerating starch synthesis and inhibiting starch degradation in drought‐tolerant mulberry GY2024.

##### ABA biosynthesis‐related genes

**FIGURE 4:**
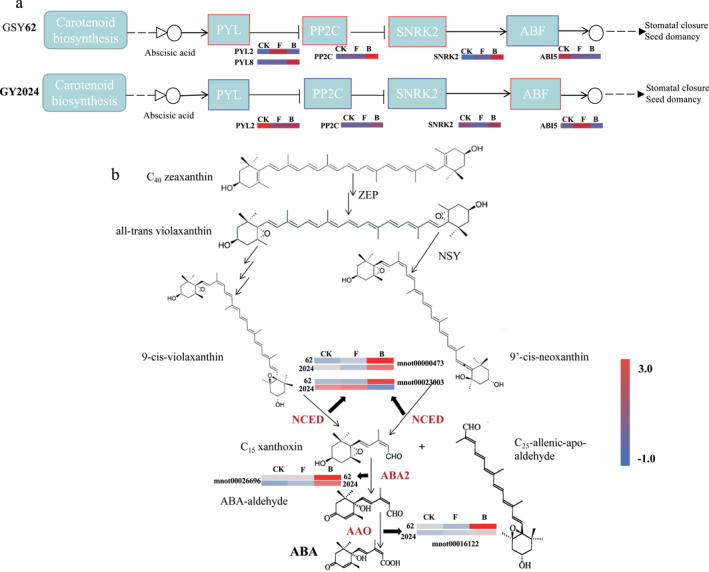
Simplified mechanistic model of the key DEGs in GSY62 and GY2024 and the ABA (a) direct and (b) indirect biosynthesis pathways. The blue box represents gene downregulation and the red box represents gene upregulation. Each coloured cell represents the average log2 (TPM) value according to the colour scale; red represents high expression and blue represents low expression.

Plant hormones are active organic compounds that resist adverse external environments during plant growth and development, with the content of abscisic acid (ABA) being one of the important indicators of plant drought resistance. In GSY62, there were two DEGs associated with encoding PYL(PYL2/PYL8), whose expression levels were upregulated by 20.0 and 3.6 times under drought stress, respectively. In GY2024, there was only one DEG associated with encoding PYL (PYL2) and its expression level was down regulated by 4.1 times under drought stress. Interestingly, three DEGs encoding PP2C, SNRK2, and ABF were the same in GSY62 and GY2024, but their expression patterns were indeed opposite. For example, the DEG encoding ABF (ABI5) was upregulated by 3.8 in GY2024 and downregulated by 5.9 in GSY62 (Figure 4a). The above results indicated that the synthesis and metabolism of ABA in mulberry were affected by drought and the responses of key enzyme‐related genes in the ABA signalling pathway differed in mulberry varieties with different drought resistances.

The carotenoid biosynthesis pathway is the main ABA synthesis pathway in plants. Therefore, the expression of key enzyme‐encoding genes related to carotenoid biosynthesis was also detected. These genes included 9‐cis‐epoxycarotenoid dioxygenase (NCED), short‐chain dehydrogenase (ABA2) and ABA aldehyde oxidase (AAO). The results indicated that the expression of NCED was significantly upregulated in both GSY62 and GY24 after stress, but the expression level of NCED in GY2024 was 16 times higher than that in GSY62 in the early stage of drought stress. Overall, the upregulation of the above carotenoid biosynthesis‐related enzyme‐related genes in both the GSY62 and GY2024 strains promoted the accumulation of ABA and contributed to enhancing the drought resistance of mulberry (Figure 4b).

##### Flavonoid biosynthesis pathway‐related genes

**FIGURE 5:**
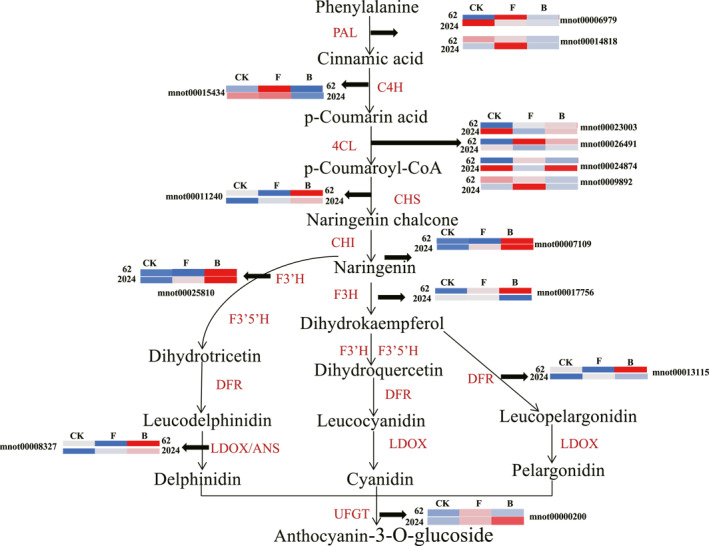
Simplified mechanistic model of the key DEGs in GSY62 and GY2024 in the flavonoid biosynthesis pathway. Each coloured cell represents the average log2 (TPM) value according to the colour scale; red represents high expression and blue represents low expression.

Previous studies have reported that secondary metabolite content plays a crucial role in enhancing plant drought resistance. Therefore, the expression of genes associated with flavonoids and anthocyanin biosynthesis in GY2024 and GSY62 under drought stress were also detected, including phenylalanine ammonia lyase (PAL), cinnamate 4‐hydroxylase (C4H), 4‐coumaroyl CoA ligase (4CL), chalcone synthase (CHS), chalcone isomerase (CHI), flavanone 3′‐hydroxylase (F3′H), dihydroflavonol‐4‐reductase (DFR) and anthocyanidin 3‐O‐glucosyltransferase (LDOX). Among these, the expression of the most vital enzyme‐related genes was significantly upregulated. Additionally, the response of the same gene in different mulberry varieties also exhibited differential expression. In GSY62, the gene mnot00017756 (encoding F3H) was upregulated 7.9‐fold after drought stress treatment. However, the expression level of mnot00017756 showed no significant difference in GY2024. Additionally, the expression of anthocyanin biosynthesis‐related genes was significantly higher in GY2024 than in the GSY62 strain (Figure 5). The above results indicated that the drought stress conditions significantly enhanced the metabolic activity of flavonoid biosynthesis in both the GSY62 and GY2024 strains.

#### 3.1.5 Identification and expression analysis of transcription factors related to drought stress

To identify the hypothetical regulators of drought resistance, a total of 478, 560, 260 and 368 TFs were identified in 62F, 62B, 2024F and 2024B, respectively (Table S8). Among these, the most numerous TFs were MYB/MYB‐related (MYB domain proteins), followed by ERF (ethylene responsive factor), bHLH (basic helix‐loop‐helix), WRKY (WRKY proteins), NAC (NAM/ATAF/CUC) and bZIP (basic region‐leucine zipper). The results showed that 55.13% (43/78) and 52.17% (43/78) of the MYB family TFs were upregulated in 62F and 62B, respectively. In contrast, only 48.98% (24/49) and 35.60% (21/59) of MYB family TFs were upregulated in 2024F and 2024B, respectively. Notably, only 25.64% (10/39) of the NAC family was upregulated in 62F but 75% (18/24) was in 2024F. Additionally, the response of the same TFs in different mulberry varieties also varied. For example, the expression level of the gene mnot00002444 (encoding MYB4) was upregulated 136.4‐fold in GSY62 and there was no significant difference in GY2024. Furthermore, the expression level of the gene mnot00000735 (encoding NAC‐72) was upregulated 724.0‐fold in GSY62 but only 212.8‐fold in GY2024. The above differential expression of TF genes in GSY62 and GY2024 suggested that these TFs were involved in the regulation of drought stress in mulberry trees, and the TFs of the MYB and NAC families may be the pivotal TFs underpinning the difference in drought resistance between the GSY62 and GY2024 strains.

#### 3.1.6 Validation of gene expression using qRT‐qPCR

**FIGURE 6:**
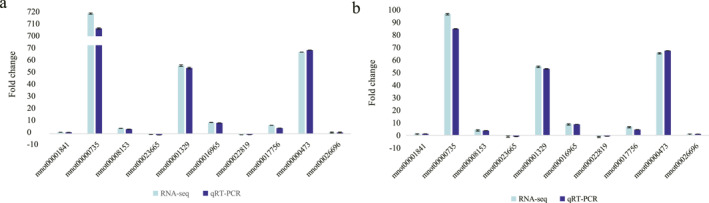
qRT‐PCR verification of 10 genes related to key metabolic pathways in (a) GSY62 and (b) GY2024.

Based on the results, twelve drought resistance‐related genes were selected for transcriptome sequencing validation using qRT‐PCR (Table S9). These genes were involved in the processes of starch and sucrose metabolism, flavonoid biosynthesis, and ABA signal transduction and also contained the key TFs. The qRT‐PCR results demonstrated that all twelve selected genes were differentially expressed between the GSY62 and GY2024 strains (*p* < 0.05) and the expression profiles of these genes displayed similar patterns to the TPM (transcripts per million reads) values found in transcriptome sequencing under the corresponding treatments (Figure 6a,b). The above results indicated that this study obtained credible analysis results based on reliable RNA‐seq data.

### 3.2 Metabolome analysis of the GSY62 and GY2024 strains

**FIGURE 7:**
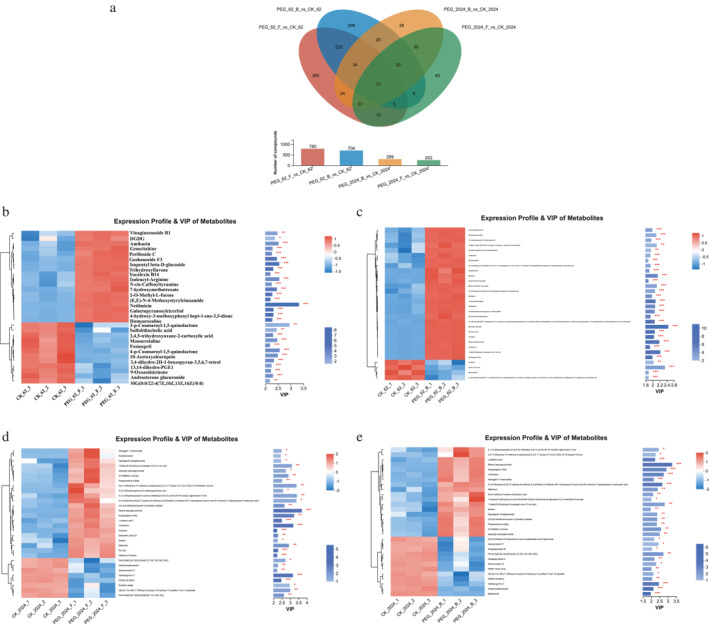
Wayne chart, expression profile and VIP of DEMs in GSY62 and GY2024. (a) Wayne chart of DEMs in GSY62 and GY2024. (b) Expression profile and VIP of DEMs in 62F vs. CK. (c) Expression profile and VIP of DEMs in 62B vs. CK. (d) Expression profile and VIP of DEMs in 2024F vs. CK. (e) Expression profile and VIP of DEMs in 2024B vs. CK. Each column represents a sample, with the sample name below. Each row represents a metabolite and the colour represents the relative expression level of that metabolite in a group of samples. The corresponding relationship between the colour gradient and numerical size is shown in the gradient colour block. The length of the VIP bar indicates the contribution value of the metabolite to the difference between the two groups. The bar colour indicates the significance of the difference in metabolites between two groups of samples (*p*‐value); the smaller the *p*‐value, the darker the colour. * indicates *p* < 0.05, ** indicates *p* < 0.01 and *** indicates *p* < 0.001.

In this study, a non‐targeted quantitative analysis of metabolites in 18 samples was also conducted. A total of 790, 704, 252 and 299 DEMs were identified in 62F, 62B, 2024F and 2024B, including 459, 448, 126 and 172 upregulated metabolites and 331, 256, 126 and 127 downregulated metabolites, respectively (Table [Link], [Link], [Link], [Link]). The Wayne diagram showed that there were 77 common DEMs among the four stress treatments (Figure 7a). Furthermore, the OPLS‐DA plot was used as the supervised model (7‐fold cross validation) and VIP analysis using the variable projection importance of the first principal component identified important differential metabolites between four comparison groups: 62F vs. CK, 62B vs. CK, 2024F vs. CK and 2024B vs. CK. From Figure S2, it can be seen that each group of samples and biologically duplicated samples clustered together with a 95% confidence level. In addition, the values of R2X, R2Y and Q2 were all close to 1, indicating that the OPLS‐DA model has a strong predictive ability, ensuring the reliability of screening differential metabolites through VIP values of metabolites in OPLS‐DA analysis. VIP value analysis showed that the metabolites in the GSY62 and GY2024 strains showed specific expression patterns.

The DEMs in the GSY62 were closely related to carbohydrate metabolism‐related pathways, such as starch and sucrose metabolism (sucrose, proline, and isoleucyl‐arginine) and the biosynthesis of other secondary metabolites mainly involved the wax, phenylpropane, flavonoid and terpenoid biosynthesis pathways rutin, ginsenoside F1, methyl jasmonate, quercetin 3‐O‐glucoside, p‐Salicylic acid, catechin, marmesinin rutinoside, 2‐O‐methyl‐L‐fucose (Figure 7b,c). The DEMs in GY2024 were closely related to carbohydrate metabolism‐related pathways, such as starch and sucrose metabolism (amylose, proline‐histidine). This result was consistent with the conclusion drawn from transcriptome analysis that drought accelerates starch synthesis and inhibits starch degradation in GY2024 (Figure 7d,e). Notably, porphobilinogen—a phototoxin, neurotoxin and endotoxin—can cause cell damage. After drought stress, its levels significantly increased in GSY62 and remained stable in GY2024. This was consistent with the results of drought resistance identification.

### 3.3 Interactive analysis of the transcriptome and metabolome of GSY62 and GY2024

To better investigate the mechanisms of drought resistance in mulberry, an interactive analysis of the transcriptome and metabolome data was performed.

#### 3.3.1 Correlation network analysis of the transcriptome and metabolome of GSY62 and GY2024

**FIGURE 8:**
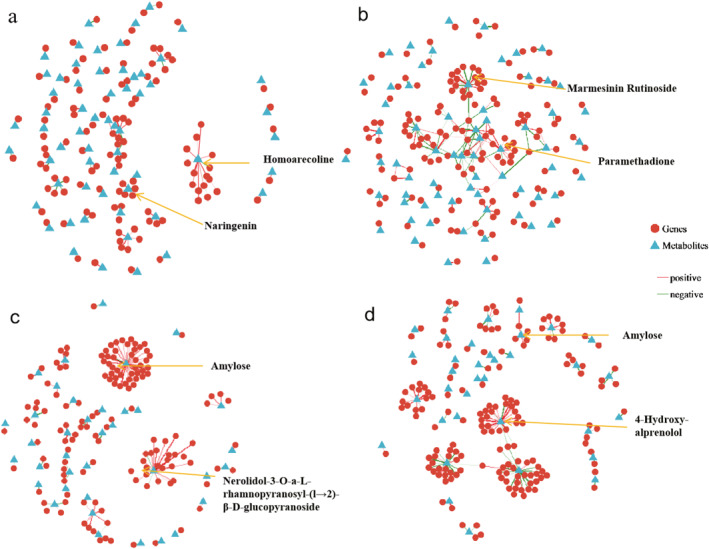
Diagram of the correlation network between metabolites and genes. The triangles represent metabolites, the circles represent genes, the lines represent correlation coefficients, the red lines represent positive correlations (correlation coefficient >0) and the green lines represent negative correlations (correlation coefficient <0). The thickness of a line corresponds to the absolute value of the correlation coefficient; the thicker the line, the greater the absolute value of the correlation coefficient and the thinner the line, the smaller the absolute value of the correlation coefficient.

After calculating the correlation coefficients between metabolites and genes, a network diagram was used to display the intermolecular interaction relationships, thus enabling the shared metabolic pathways of DEGs and DEMs to be determined (Figure 8). And the correlation network table included information such as gene_name, metabolite_name, correlation coefficient, *p* value and so on (Tables [Link], [Link], [Link], [Link]). In GSY62F, homoarecoline was associated with the most DEGs, with 19 genes, followed by 5 naringenin, with 6 DEGs associated (Figure 8a). In GSY62B, marmesinin rutinoside was associated with the most DEGs, with 15 genes, followed by Paramethadione, with 12 DEGs associated (Figure 8b). The DEGs/DEMs in GSY62 were mainly related to phenolic substances. However, the DEGs/DEMs in GY2024 were more likely to be related to terpenoids and starch (Figure 8c,d). For example, starch as a metabolite appeared in both GY2024F and GY2024B and was associated with a significant number of DEGs, especially in GY2024F where 60 DEGs were associated.

#### 3.3.2 KEGG annotation analysis on the DEGs and DEMs of GSY62 and GY2024

**FIGURE 9:**
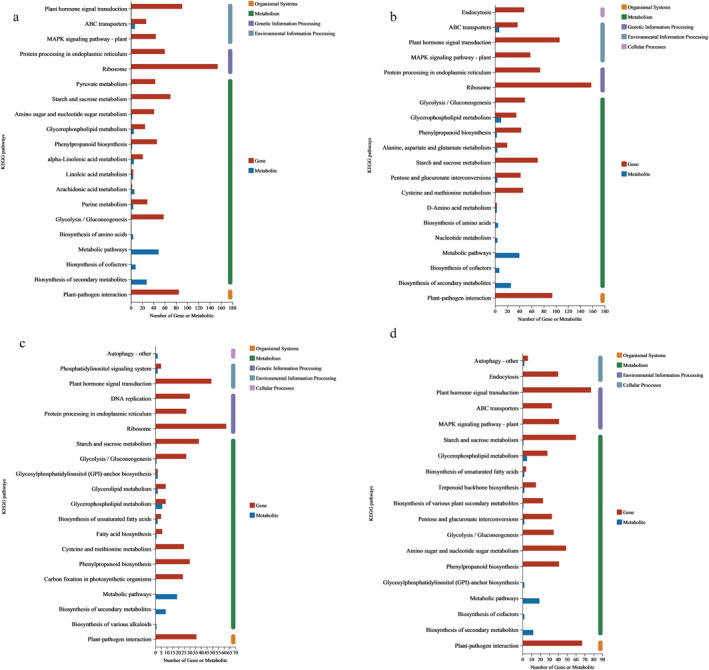
Histogram of KEGG annotation analysis of DEGs and DEMs between the four comparison groups: (a) 62F vs. CK, (b) 62B vs. CK, (c) 2024F vs CK, and (d) 2024B vs. CK. The horizontal axis represents the number of genes or metabolites in the pathway, where the blue band represents the number of genes annotated into the pathway, the red band represents the number of metabolites annotated into the pathway, and the vertical axis represents the KEGG pathway name.

To further identify the DEGs and DEMs of interest, we performed KEGG annotation on the DEGs and DEMs separately. There were a total of 11 pathways annotated to both DEGs and DEMs in GSY62F, namely plant hormone signal transduction, ABC transporters, starch and sucrose metabolism and so on (Figure 9a). In GSY62B, a total of 9 pathways were annotated for both DEGs and DEMs. These pathways included glycerophospholipid metabolism, ABC transporters, phenylpropanoid biosynthesis, and several others (Figure 9b). Regarding GY2024F, a count of 8 pathways were found to be relevant to both DEGs and DEMs. These pathways encompassed glycerophospholipid metabolism, phosphatidylinositol signalling system, starch and sucrose metabolism, as well as a number of other pathways (Figure 9c). Within GY2024B, only 7 pathways in total were annotated in relation to both DEGs and DEMs. Such pathways included glycerophospholipid metabolism, autophagy (other), and starch and sucrose metabolism (Figure 9d). Notably, the DEMs annotated in the starch and sucrose metabolism pathway were identified as sucrose in GSY62, whereas in GY2024, the annotation corresponded to amylose.

## 4 DISCUSSION

Previous studies have reported that the yield of mulberry decreases by 50%–60% when grown under drought–stress conditions. Fortunately, mulberry has demonstrated excellent adaptability to drought stress. Transcriptomic and metabonomic studies can provide insights into the molecular regulation of mulberry drought tolerance and options for prospecting drought tolerance‐linked genes. In this study, two mulberry varieties with different drought resistance—GSY62 and GY2024—were selected to clarify the molecular mechanism of mulberry drought tolerance using an integrative analysis of transcriptome sequencing and metabolomics. Other results indicated that the drought resistance of mulberry was mainly related to carbohydrate metabolism, energy metabolism, secondary metabolite biosynthesis and plant hormones. These biological processes can maintain the osmotic homoeostasis and structural integrity of mulberry cells under water stress while playing an important role in maintaining necessary life activities and signal transduction.

### 4.1 Starch synthesis and sucrose degradation enhance plant drought tolerance

The genes encoding sucrose phosphate synthase 4 and sucrose phosphatase 2 were downregulated in GY2024 but upregulated in GSY62. Additionally, the gene encoding fructose kinase‐7 was upregulated in GY2024 with an expression abundance that was higher than in GSY62. The genes encoding key enzymes for starch biosynthesis were upregulated in GY2024, whereas the key enzyme‐related genes encoding dextrin and maltose were downregulated. The above results indicated that the synthesis and metabolism of sugar in mulberry were affected by drought, with drought stress reducing the synthesis of sucrose in drought‐resistant mulberry and accelerating the degradation of sucrose. In contrast, drought stress accelerated starch synthesis and inhibited starch degradation, a result that was consistent with the metabolomic finding of a significant accumulation of amylose in GY2024 under drought stress. Similar results have also been reported that drought altered the abundance of 284 proteins, and these proteins were enriched in aspects such as photosynthesis, amino acid, sugar and starch metabolism, as well as redox regulation and reduced starch accumulation under drought conditions, leading to soluble sugar starvation at the end of the night, which in turn inhibited the leaf growth rate, indicating that starch biosynthesis contributes to maintaining leaf growth under drought stress and promotes carbon uptake during the recovery process in maize [25] It has been reported that, by separating sucrose, this pathway provides intermediates and energy sources [26, 27]. Briefly, mulberry provides energy sources for other necessary physiological activities by promoting the catabolism of sucrose and suppressing its synthetic metabolism. The findings of the present study showed that mulberry acts in a manner that is similar to other plants. However, the synthesis and decomposition of starch varies among different plants. For example, drought stress accelerates the synthesis of starch in maize and mulberry but inhibits the anabolism of starch in other plants [28, 29]. For example, studies have found that under drought conditions, the starch content in citrus decreases while the content of soluble sugars increases significantly. This process is closely related to the upregulation of the expression of the *β*‐Amylase 3 (BAM3) gene and the increase in amylase activity [30].

### 4.2 Abscisic acid synthesis pathway enhances plant drought tolerance

ABA can control the closure of plant stomata, reducing plant growth. Drought, cold, high temperatures and other stress conditions can rapidly increase ABA levels in plants, enhancing their stress resistance. ABA can induce the biosynthesis of certain enzymes and increase plant cold resistance, salt resistance and drought resistance. Therefore, ABA is referred to as a stress hormone [31, 32, 33]. PP2C is a negative regulatory factor for ABA [34]. In this study, the key enzyme‐related gene encoding PP2C was upregulated in GSY62, while in GY2024 was downregulated. Moreover, ABA Insensitive 5 was significantly upregulated in GY2024, which may have been directly related to drought resistance. The expression of PP2C genes was significantly downregulated and the expression of protein ABA Insensitive 5 gene in the ABF gene family was also significantly upregulated. Furthermore, genes including NCED, ABA2 and AAO were significantly upregulated in both the GSY62 and GY2024 strains and the upregulation of these genes promotes the accumulation of ABA [35]. Research has shown that ABA plays an important role in the response of plants to drought stress. For example, Estrada‐Melo et al. overexpressed the tomato *NCED* (*LeNCED1*) in petunia plants. Under water stress, the transgenic plants had increased transcripts of *NCED* mRNA, elevated leaf ABA concentrations, increased concentrations of proline, and a significant increase in drought resistance [36]. The function of the *NCED* genes have been studied relatively maturely in mulberry. We have previously cloned the *NCED* genes (*MaNCED1* and *MaNCED2*) in mulberry and analysed their expression pattern under drought stress [37]. The results showed that drought stress significantly upregulated their expression level. In addition, functionally active NCED proteins were obtained, and they were found to promote the production of ABA precursor substances [38]. Furthermore, Fan et al. showed that the expression of *MaNCED1* in the root was strongly induced by stress and salt. By increasing the expression of *MaNCED1* in tobacco using overexpression, compared with the wild type, the accumulation of H_2_O_2_ and MDA was reduced, whereas the POD activity and proline content were increased in the transgenic plants after drought and salt treatment [38]. These results are consistent with the significant upregulation of *NCED*, *ABA2* and *AAO* genes under drought stress in our study.

## 5 CONCLUSION

The findings of this study indicated that carbohydrate metabolism, secondary metabolite biosynthesis and plant hormones (ABA) make a significant contribution to the drought resistance of mulberry, enabling the plants to adapt to adversity by precisely regulating the levels of various molecular pathways (Abstract Figure).

## AUTHOR CONTRIBUTIONS

**Changyu Qiu**: Funding acquisition; Resources. **Sheng Huang**: Data curation; Software. **Rongli Mo**: Methodology; Software. **Xiaomei Lu**: Formal analysis. **Yanrong Zeng**: Formal analysis. **Guangshu Zhu**: Formal analysis. **Chaohua Zhang**: Formal analysis. **Qiang Lin**: Conceptualisation; Writing—review and editing.

## CONFLICT OF INTEREST STATEMENT

The authors declare no conflicts of interest.

## Supporting information

Supporting Information S1

Supporting Information S2

Figure S1

Figure S2

Table S4

Table S5

Table S6

Table S7

Table S8

Table S9

Table S10

Table S11

Table S12

Table S13

Table S14

Table S15

Table S16

Table S17

## Data Availability

The authors confirm that the data supporting the findings of this study are available within the article [and/or] its supplementary materials.
