# Supplementary material for: Transcriptome sequencing and metabolome analysis to reveal renewal evidence for drought adaptation in mulberry
Source: IET Syst Biol. 2025 Feb 26;19(1):e70004. doi: 10.1049/syb2.70004 (PMC11865340; doi:10.1049/syb2.70004)
Supplement: Supplementary file 1 — Supporting Information S1 [file SYB2-19-e70004-s004.doc]

**Figure S1. Correlation analysis between samples: 62CK, 62F, 62B, 2024CK, 2024F, 2024B. (a) Venn diagram analysis between samples. (b) Correlation heatmap between samples (c) PCA analysis between samples.** Different colored circles in figure 1a represent genes screened based on expression levels in a sample, and numerical values represent the number of common and unique genes among different samples. When the number of sample in the parameter setting is ≤ 5, the sum of all numbers inside the circle represents the total number of genes in the sample, and the cross area of the circle represents the number of common gene in each sample. The right and bottom sides in the figure 1b represent the sample names, while the left and top sides represent the clustering situation of the samples. The different colored squares represent the high or low correlation between the two samples. The distance between each sample point in the figure S1c represents the distance of the sample. The closer the distance, the higher the similarity between the samples. The horizontal axis represents the contribution of Principal Component 1 (PC1) to the discriminative samples in the two-dimensional graph, while the vertical axis represents the contribution of Principal Component 2 (PC2) to the discriminative samples in the two-dimensional graph.

**Figure S2. OPLS-DA plots between four comparison groups: 62F vs. CK, 62B vs. CK, 2024F vs. CK and 2024B vs. CK. (a) OPLS-DA plot between 62F vs. CK. (b) OPLS-DA plot between 62B vs. CK. (c) OPLS-DA plot between 2024F vs. CK. (d) OPLS-DA plot between 2024B vs. CK.** Comp1 represents the first predicted principal component decomposition degree, and orthogonal Comp1 represents the first orthogonal component decomposition degree.The confidence ellipse represents the distribution of "real" samples within this region with a 95% confidence level; Exceeding this area can be considered as a possible abnormal sample. R2X and R2Y represent the explanatory power of the constructed model for the X and Y matrices, respectively, while R2X (cum) and R2Y (cum) represent the cumulative explanatory power; Q2 indicates the predictive ability of the model. The closer these three indicators are to 1, the more stable and reliable the model is. Q2>0.5 indicates good predictive ability, while Q2<0.5 indicates poor predictive ability.
