# Supplementary material for: Transcriptome sequencing and metabolome analysis to reveal renewal evidence for drought adaptation in mulberry
Source: IET Syst Biol. 2025 Feb 26;19(1):e70004. doi: 10.1049/syb2.70004 (PMC11865340; doi:10.1049/syb2.70004)
Supplement: Supplementary file 2 — Supporting Information S2 [file SYB2-19-e70004-s013.docx]

## Supplemental Tables

**Supplemental Table 1: The raw data and quality control data of the sample**

| Sample | Raw reads | Raw bases | Clean reads | Clean bases | Error rate(%) | Q20(%) | Q30(%) | GC content(%) |
| --- | --- | --- | --- | --- | --- | --- | --- | --- |
| CK_62_A | 42091894 | 6313784100 | 42091878 | 6296904141 | 0 | 100 | 100 | 46.37 |
| CK_62_B | 42554734 | 6383210100 | 42554726 | 6356427905 | 0 | 100 | 100 | 46.55 |
| CK_62_C | 42008102 | 6301215300 | 42008084 | 6285202861 | 0 | 100 | 100 | 46.31 |
| PEG_62_F_A | 42298252 | 6344737800 | 42298248 | 6321027977 | 0 | 100 | 100 | 46.23 |
| PEG_62_F_B | 43260142 | 6489021300 | 43260128 | 6470513267 | 0 | 100 | 100 | 46.29 |
| PEG_62_F_C | 42042014 | 6306302100 | 42042000 | 6290235160 | 0 | 100 | 100 | 46.29 |
| PEG_62_B_A | 42045288 | 6306793200 | 42045266 | 6288512628 | 0 | 100 | 100 | 45.67 |
| PEG_62_B_B | 42947398 | 6442109700 | 42947374 | 6417253540 | 0 | 100 | 100 | 45.86 |
| PEG_62_B_C | 42659274 | 6398891100 | 42659242 | 6381668289 | 0 | 100 | 100 | 45.76 |
| CK_2024_A | 43474984 | 6521247600 | 43474972 | 6503247619 | 0 | 100 | 100 | 46.68 |
| CK_2024_B | 42859104 | 6428865600 | 42859096 | 6411350874 | 0 | 100 | 100 | 46.44 |
| CK_2024_C | 43529912 | 6529486800 | 43529904 | 6518239256 | 0 | 100 | 100 | 46.38 |
| PEG_2024_F_A | 43390572 | 6508585800 | 43390560 | 6490363066 | 0 | 100 | 100 | 46.05 |
| PEG_2024_F_B | 42552332 | 6382849800 | 42552320 | 6362512351 | 0 | 100 | 100 | 46.02 |
| PEG_2024_F_C | 43402748 | 6510412200 | 43402740 | 6497617292 | 0 | 100 | 100 | 45.98 |
| PEG_2024_B_A | 43502378 | 6525356700 | 43502368 | 6506958217 | 0 | 100 | 100 | 45.86 |
| PEG_2024_B_B | 41991254 | 6298688100 | 41991248 | 6283905787 | 0 | 100 | 100 | 46.01 |
| PEG_2024_B_C | 43187732 | 6478159800 | 43187722 | 6464373018 | 0 | 100 | 100 | 45.94 |

Q20 and Q30 refer to the percentage of bases with sequencing quality above 99% and 99.9%, respectively, in total bases. Generally, Q20 is above 85% and Q30 is above 80%.

**Supplemental Table 2: Sequence alignment between Clean Reads of the sample and the designated reference genome**

| Sample | Total reads | Total mapped | Multiple mapped | Uniquely mapped |
| --- | --- | --- | --- | --- |
| CK_62_A | 42091878 | 30280059(71.94%) | 927927(2.2%) | 29352132(69.73%) |
| CK_62_B | 42554726 | 30235589(71.05%) | 935804(2.2%) | 29299785(68.85%) |
| CK_62_C | 42008084 | 30130329(71.73%) | 910559(2.17%) | 29219770(69.56%) |
| PEG_62_F_A | 42298248 | 29929832(70.76%) | 952157(2.25%) | 28977675(68.51%) |
| PEG_62_F_B | 43260128 | 30304146(70.05%) | 968669(2.24%) | 29335477(67.81%) |
| PEG_62_F_C | 42042000 | 29807534(70.9%) | 953927(2.27%) | 28853607(68.63%) |
| PEG_62_B_A | 42045266 | 29676687(70.58%) | 886526(2.11%) | 28790161(68.47%) |
| PEG_62_B_B | 42947374 | 30514422(71.05%) | 924514(2.15%) | 29589908(68.9%) |
| PEG_62_B_C | 42659242 | 29870015(70.02%) | 893912(2.1%) | 28976103(67.92%) |
| CK_2024_A | 43474972 | 30775700(70.79%) | 936780(2.15%) | 29838920(68.63%) |
| CK_2024_B | 42859096 | 30606149(71.41%) | 901868(2.1%) | 29704281(69.31%) |
| CK_2024_C | 43529904 | 30836275(70.84%) | 919719(2.11%) | 29916556(68.73%) |
| PEG_2024_F_A | 43390560 | 30134578(69.45%) | 922784(2.13%) | 29211794(67.32%) |
| PEG_2024_F_B | 42552320 | 29920015(70.31%) | 922926(2.17%) | 28997089(68.14%) |
| PEG_2024_F_C | 43402740 | 30414744(70.08%) | 929918(2.14%) | 29484826(67.93%) |
| PEG_2024_B_A | 43502368 | 30189184(69.4%) | 824215(1.89%) | 29364969(67.5%) |
| PEG_2024_B_B | 41991248 | 29789812(70.94%) | 808702(1.93%) | 28981110(69.02%) |
| PEG_2024_B_C | 43187722 | 30107535(69.71%) | 812132(1.88%) | 29295403(67.83%) |

**Supplemental Table 3**: **Statistical table of new transcript types.**

| Class_code | Description | Number |
| --- | --- | --- |
| = | Complete match of intron chain | 26010 |
| i | Transfrag falling entirely within a reference intron | 292 |
| j | Potentially novel isoform (fragment):at least one splice junction is shared with a reference transcript | 16299 |
| o | Generic exonic overlap with a referfence transcript | 1354 |
| u | Unknown, intergenic transcript | 2945 |
| x | Exonic overlap with reference on the opposite strand | 504 |
| c | Contained | 0 |
| e | Single exon transfrag overlapping a reference exon and at least 10 bp of a reference intron,indiction a possible pre-mRNA fragment | 0 |
| p | Possible polymerase run-on fragment(within 2Kbases of a reference transcript | 0 |
| r | Repeat. Currently determined by looking at the soft-masked reference sequence and applied to transcripts where at least 50% of the bases are lower case | 0 |
| s | An intron of the transfrag overlaps a reference intron on the opposite strand(likely due to read mapping errors | 0 |
| . | Tracking file only,indicates multiple classifications | 0 |
