# Supplementary figures and images for: Transcriptome sequencing and metabolome analysis to reveal renewal evidence for drought adaptation in mulberry
Source: IET Syst Biol. 2025 Feb 26;19(1):e70004. doi: 10.1049/syb2.70004 (PMC11865340; doi:10.1049/syb2.70004)

Venn

a

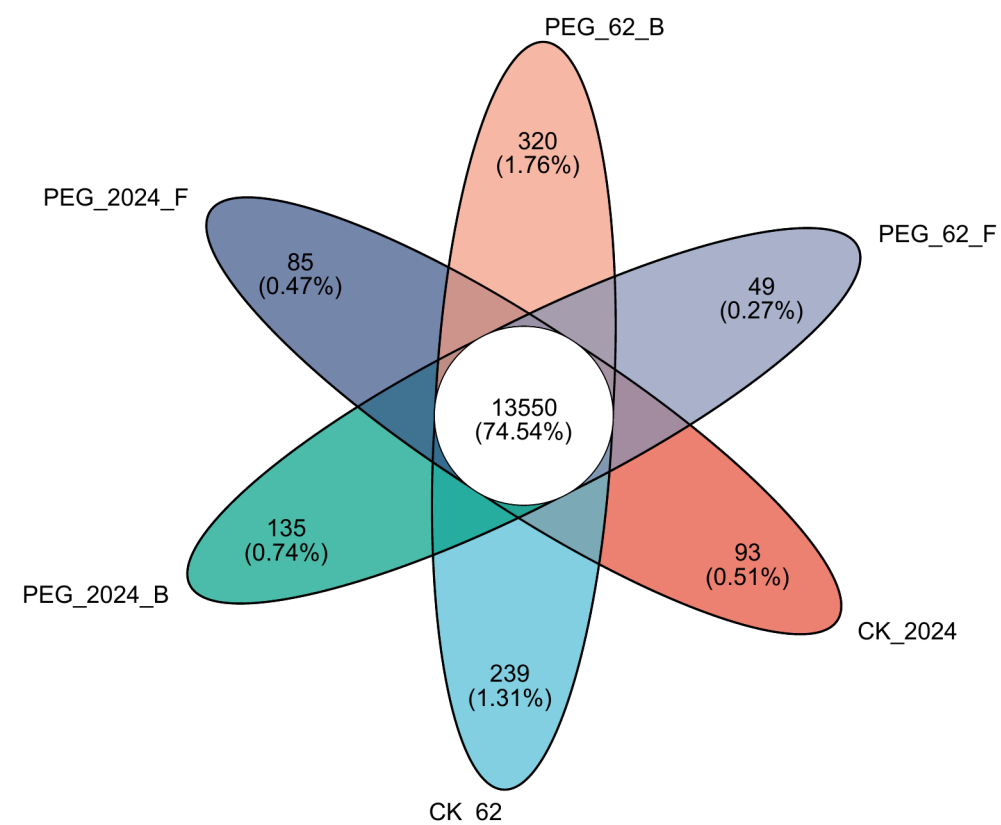

b

Correlation between samples

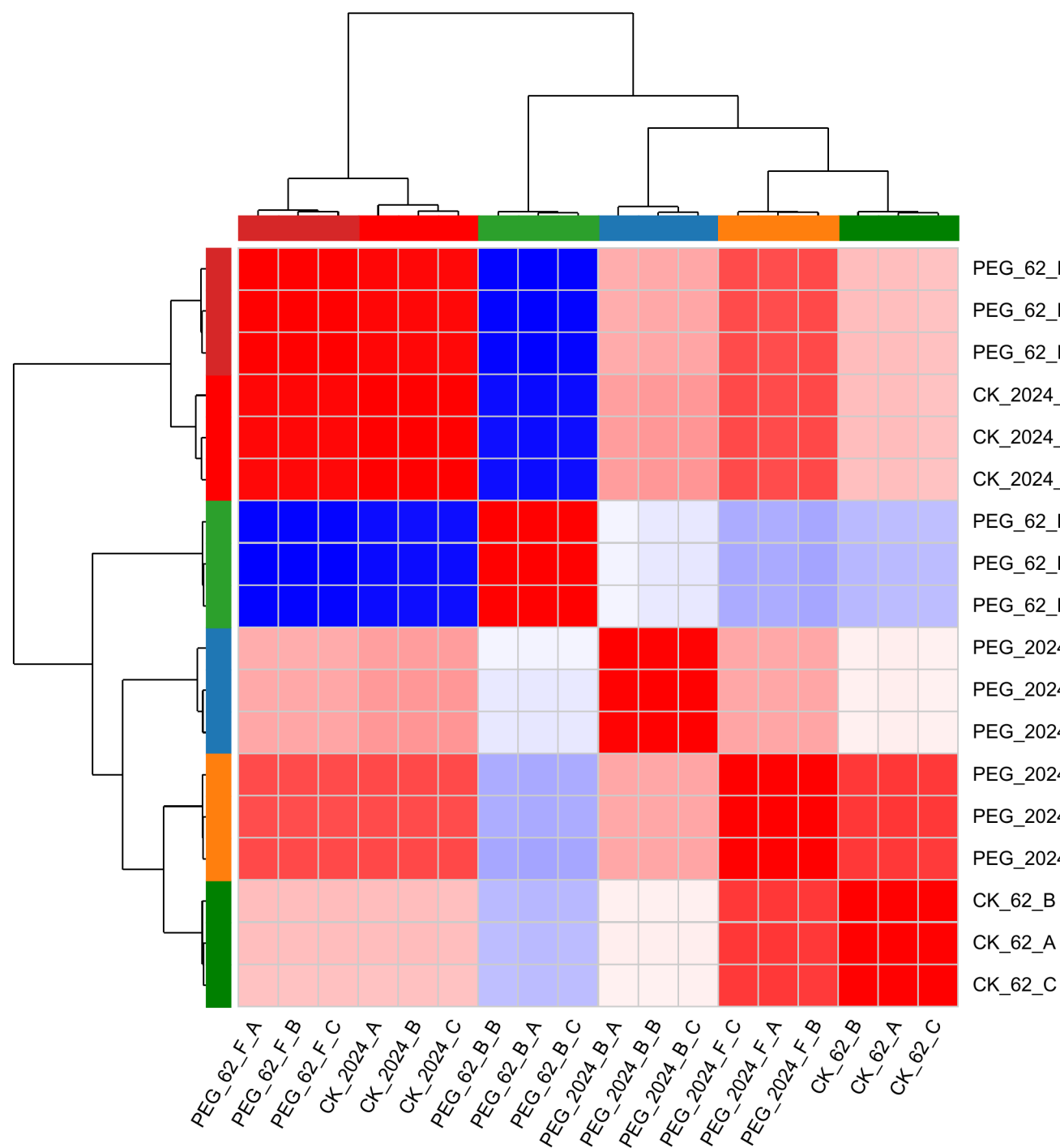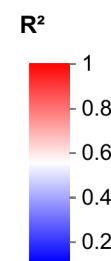

c

PCA analysis

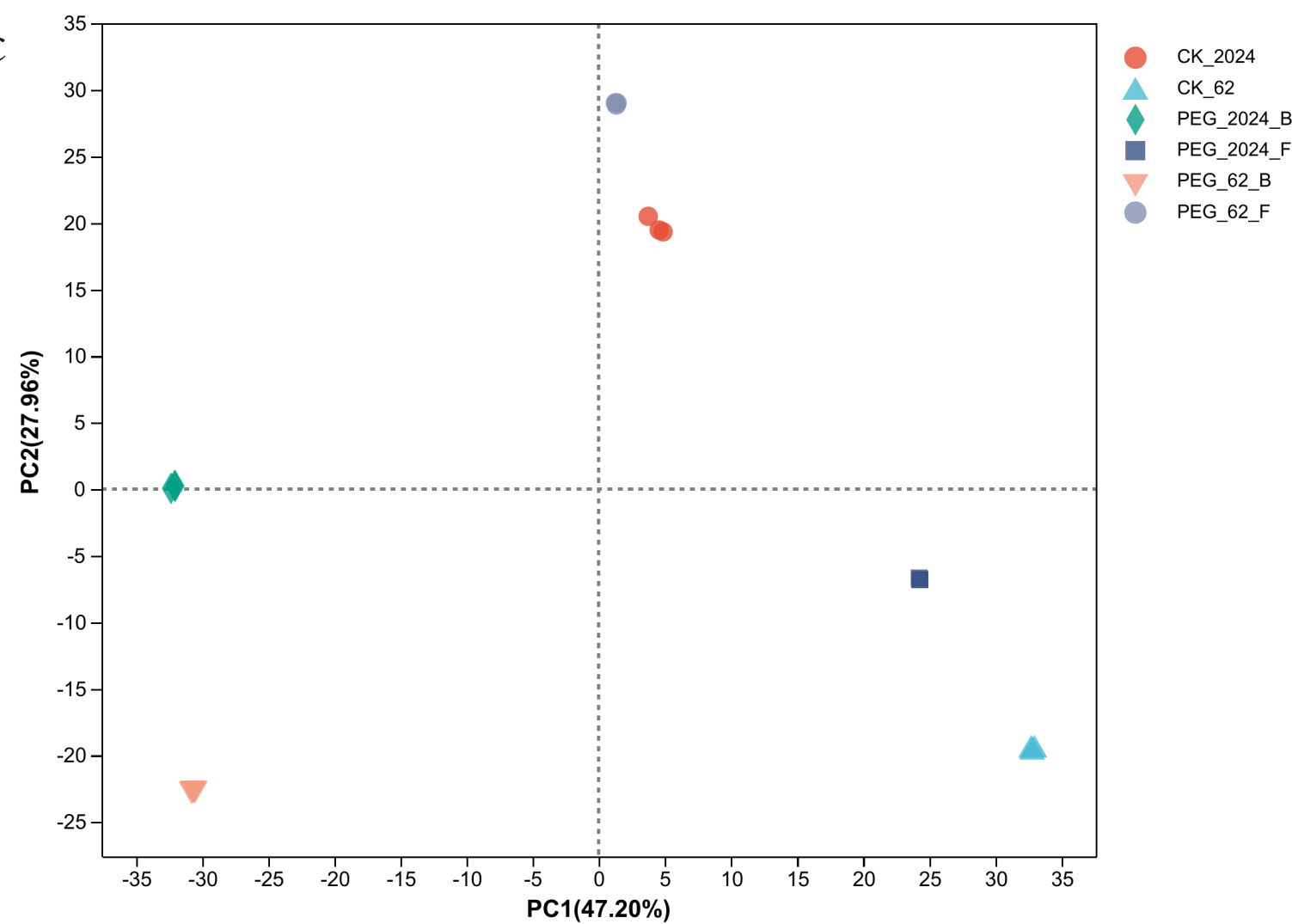

Supplement: Supplementary file 3 — Figure S1 [file SYB2-19-e70004-s010.pdf]

a

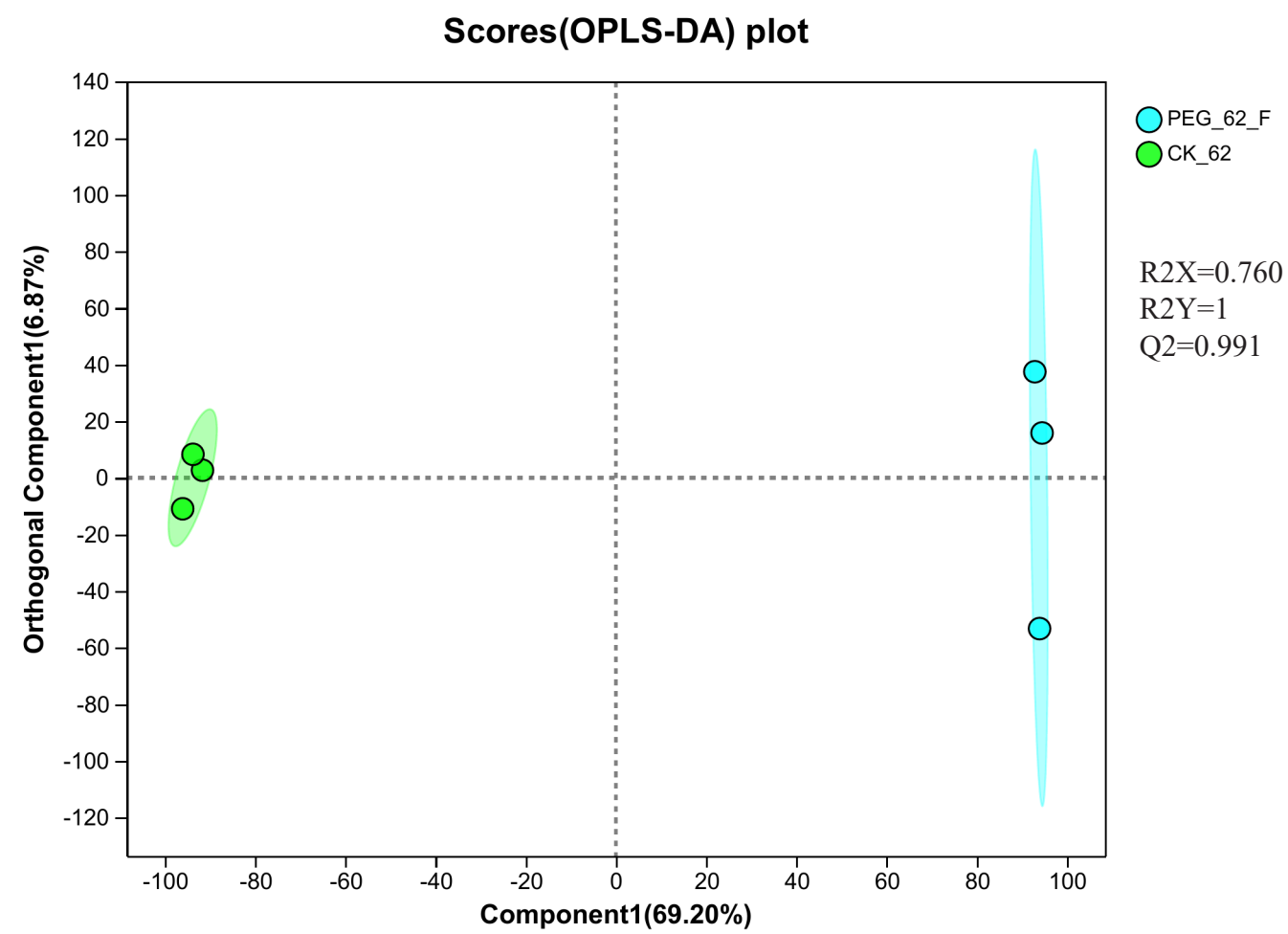

b

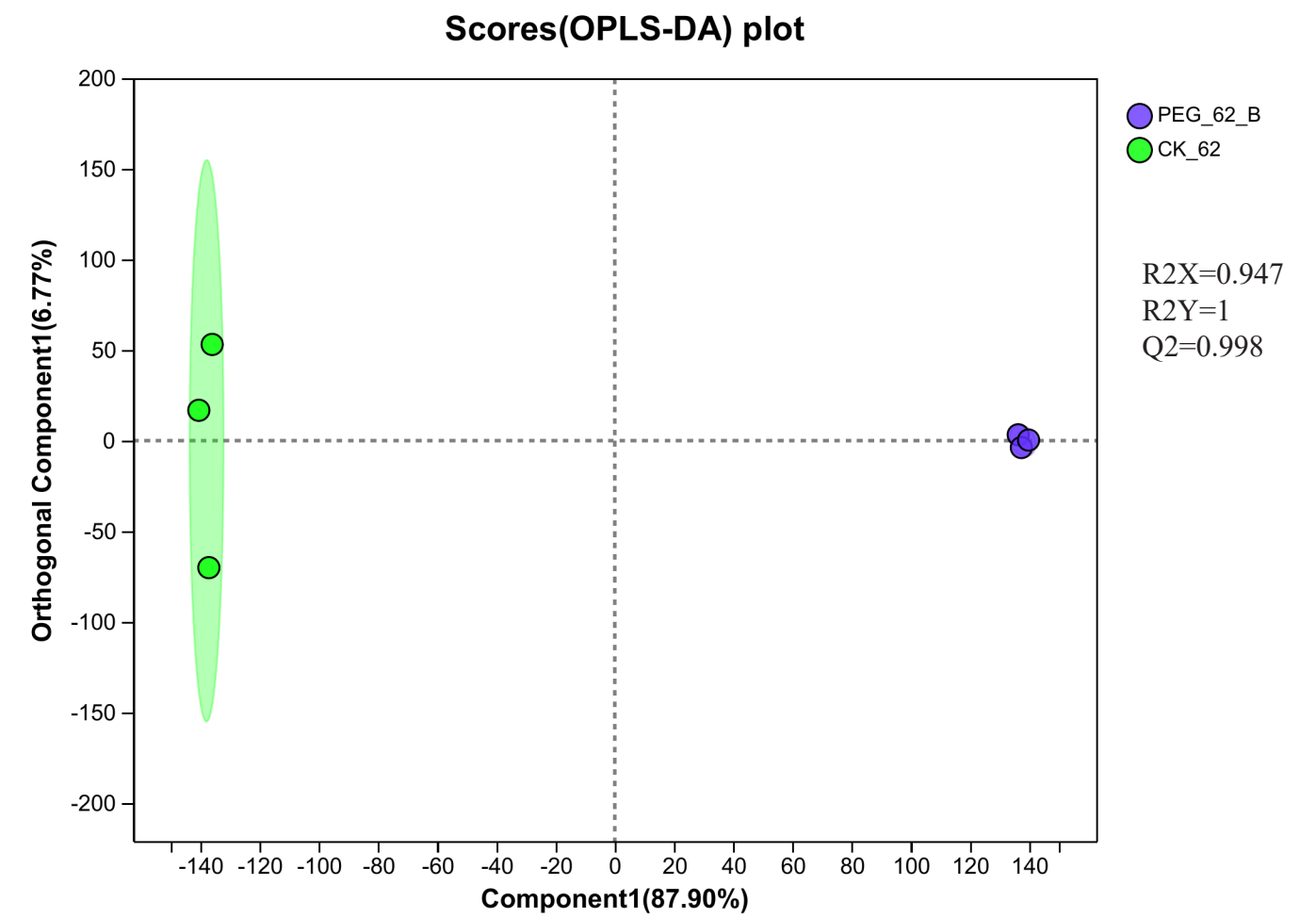

c

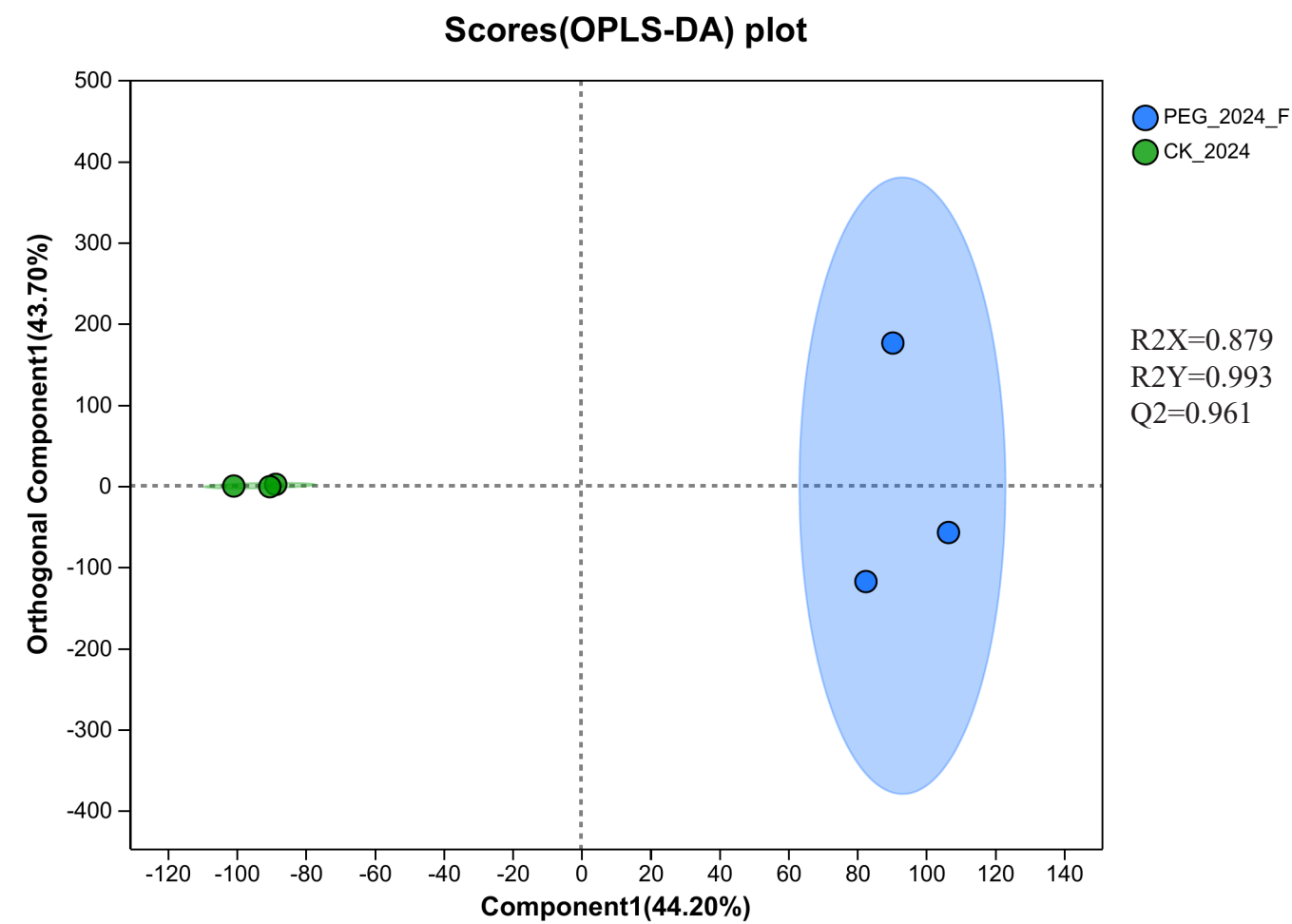

d

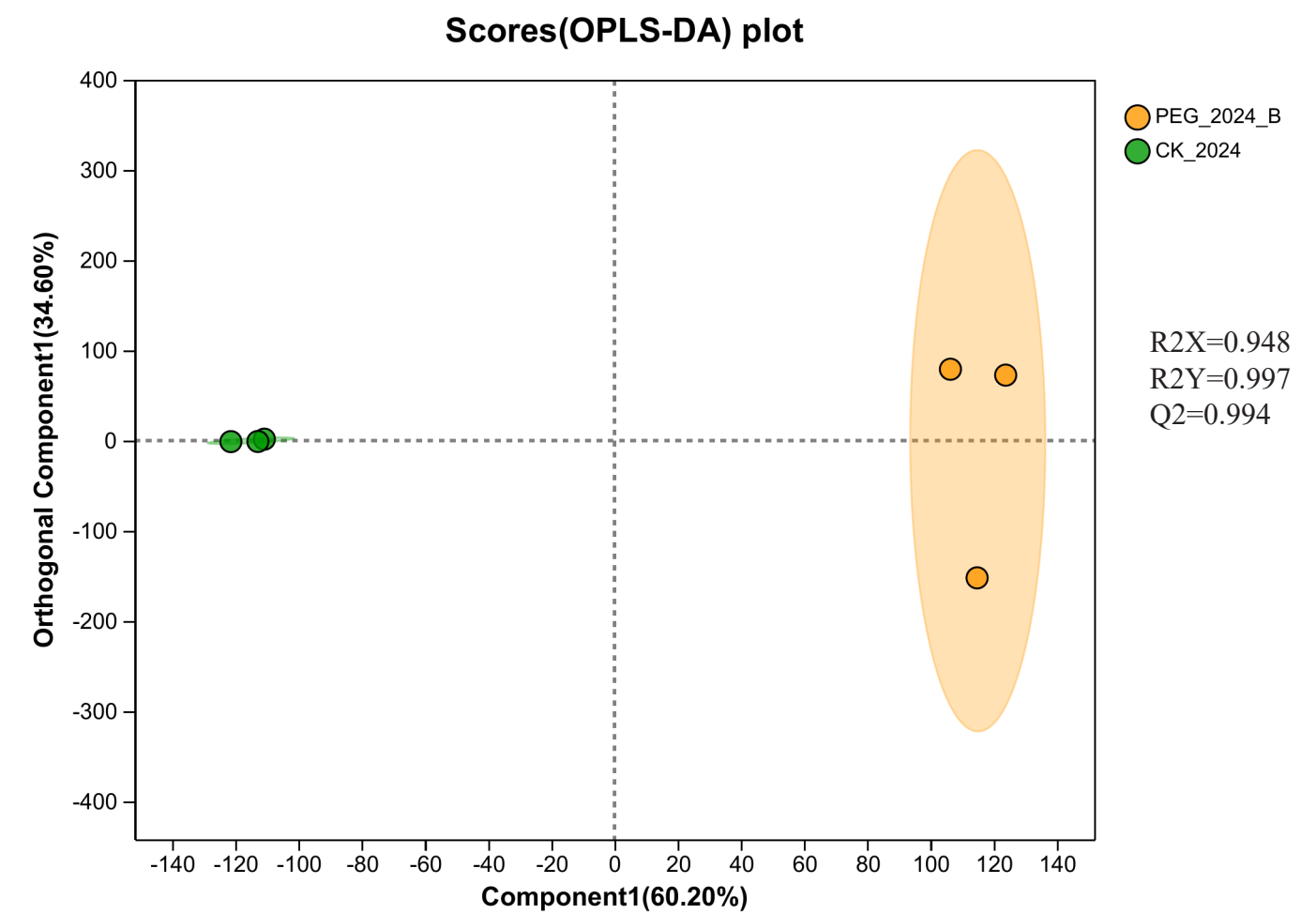

Supplement: Supplementary file 4 — Figure S2 [file SYB2-19-e70004-s011.pdf]
