# Supplementary material for: Transcriptome sequencing and metabolome analysis to reveal renewal evidence for drought adaptation in mulberry
Source: IET Syst Biol. 2025 Feb 26;19(1):e70004. doi: 10.1049/syb2.70004 (PMC11865340; doi:10.1049/syb2.70004)
Supplement: Supplementary file 9 — Table S8 [file SYB2-19-e70004-s007.doc]

Supplementary Table 8 Statistical Table of Transcription Factor Families

| Family | 62FvsCK | 62BvsCK | 2024FvsCK | 2024BvsCK |
| --- | --- | --- | --- | --- |
| AP2 | 8 | 8 | 5 | 4 |
| ARF | 5 | 7 | 2 | 5 |
| B3 | 19 | 19 | 9 | 14 |
| C2H2 | 8 | 10 | 5 | 6 |
| C3H | 8 | 6 | 2 | 8 |
| CAMTA | 0 | 2 | 0 | 2 |
| CO-like | 5 | 5 | 3 | 4 |
| CPP | 4 | 2 | 4 | 2 |
| DBB | 8 | 6 | 3 | 3 |
| Dof | 14 | 9 | 2 | 9 |
| E2F/DP | 5 | 3 | 4 | 3 |
| EIL | 1 | 4 | 0 | 1 |
| ERF | 39 | 52 | 24 | 29 |
| GATA | 9 | 8 | 5 | 6 |
| GRAS | 18 | 19 | 8 | 13 |
| GRF | 8 | 9 | 8 | 8 |
| GeBP | 0 | 1 | 0 | 1 |
| HB-other | 39 | 38 | 22 | 16 |
| HD-ZIP | 8 | 11 | 4 | 3 |
| HSF | 12 | 15 | 4 | 8 |
| LBD (AS2/LOB) | 7 | 7 | 5 | 9 |
| LSD | 0 | 1 | 0 | 1 |
| MIKC | 9 | 12 | 6 | 7 |
| MYB/MYB_related | 78 | 92 | 49 | 59 |
| M_type | 15 | 18 | 7 | 8 |
| NAC | 27 | 38 | 12 | 25 |
| NF-YA | 1 | 6 | 1 | 4 |
| NZZ/SPL | 1 | 2 | 0 | 1 |
| SBP | 6 | 11 | 8 | 10 |
| SRS | 2 | 2 | 3 | 2 |
| TCP | 6 | 10 | 4 | 6 |
| WRKY | 29 | 40 | 15 | 32 |
| YABBY | 3 | 4 | 2 | 4 |
| ZF-HD | 7 | 9 | 6 | 5 |
| bHLH | 40 | 40 | 22 | 34 |
| bZIP | 19 | 25 | 4 | 16 |
| FAR1 | 3 | 2 | 0 | 0 |
| NF-X1 | 1 | 0 | 0 | 0 |
| Nin-like | 3 | 2 | 2 | 0 |
| RAV | 1 | 0 | 0 | 0 |
| TALE | 1 | 1 | 0 | 0 |
| Whirly | 1 | 2 | 0 | 0 |
| BBR-BPC | 0 | 1 | 0 | 0 |
| BES1 | 0 | 1 | 0 | 0 |
| 合计 | 478 | 560 | 260 | 368 |
