# Supplementary material for: Transcriptome sequencing and metabolome analysis to reveal renewal evidence for drought adaptation in mulberry
Source: IET Syst Biol. 2025 Feb 26;19(1):e70004. doi: 10.1049/syb2.70004 (PMC11865340; doi:10.1049/syb2.70004)
Supplement: Supplementary file 10 — Table S9 [file SYB2-19-e70004-s005.doc]

**Supplemental Table 9: qRT-PCR primers of 10 genes related to key metabolic pathways**

| Numbers | Genes | Annotations | Primers | Primer sequences (5' to 3') |
| --- | --- | --- | --- | --- |
| 1 | mnot00001841 | sucrose-phosphate synthase 4 | F | AGGTTTGAAGGCGAAGAC |
|  |  |  | R | CTGATAAGGACGATGTAGAGG |
| 2 | mnot00000735 | NAC domain-containing protein 72 | F | GGATGAGTGGGTGCTGTG |
|  |  |  | R | CCGACGGTTCCTTGGT |
| 3 | mnot00008153 | soluble starch synthase 1 | F | AAGGATGCCCGAAGT |
|  |  |  | R | CCAATGCTCCATACCA |
| 4 | mnot00023665 | 1,4-alpha-glucan-branching enzyme 3 | F | CCAGAAGACGCATACAA |
|  |  |  | R | AATCGGCTGCTAACG |
| 5 | mnot00001329 | protein phosphatase 2C 77 | F | TCCACCTCGCTTGTTG |
|  |  |  | R | CATCGCAGTTGCTTCC |
| 6 | mnot00016965 | Serine/threonine-protein kinase SAPK2 | F | ATGGAACGCTATGAGAT |
|  |  |  | R | CAAAGTTTCCTGACCC |
| 7 | mnot00022819 | ABSCISIC ACID-INSENSITIVE 5 | F | GGTCGGAAGCGGATAA |
|  |  |  | R | GGTCGGAAGCGGATAA |
| 8 | mnot00017756 | flavanone 3-hydroxylase 1 | F | CGAGTTCTTCGCCTTGC |
|  |  |  | R | GAGCCTCCTTATCTAATCCC |
| 9 | mnot00000473 | 9-cis-epoxycarotenoid dioxygenase 1 | F | CCACTACCACTCCGACTTAC |
|  |  |  | R | CTGGACGAGCCGCTTA |
| 10 | mnot00026696 | xanthoxin dehydrogenase | F | GGCATTGGTCACAGGG |
|  |  |  | R | TCTACAGCACGGCTAACAT |
